# Human infections with novel reassortant H5N6 avian influenza viruses in China

**DOI:** 10.1038/emi.2017.38

**Published:** 2017-06-07

**Authors:** Ye Zhang, Minmei Chen, Yiwei Huang, Wenfei Zhu, Lei Yang, Lidong Gao, Xiaodan Li, Fuyin Bi, Chaoyang Huang, Ning Kang, Hengjiao Zhang, Zi Li, Hong Bo, Dayan Wang, Yuelong Shu

**Affiliations:** 1 National Institute for Viral Disease Control and Prevention, Collaboration Innovation Center for Diagnosis and Treatment of Infectious Diseases, Chinese Center for Disease Control and Prevention, Key Laboratory for Medical Virology, National Health and Family Planning Commission, Beijing 102206, China; 2 Guangxi Center for Disease Prevention and Control, Nanning 530028, China; 3 Hunan Provincial Center for Disease Control and Prevention, Changsha 410005, China

**Dear Editor,**

In May 2014, China officially reported the first human infection with highly pathogenic H5N6 subtype avian influenza virus (AIV) in Sichuan Province.^1^ Through 2nd Feb, 17 H5N6 cases were documented (http://www.who.int/en/), and H5N6 viruses isolated from both humans and poultry showed marked reassortment.^2, 3, 4^ We have previously identified three major reassortant H5N6 viruses, including reassortants A, B and C.^4^ The viruses with these three reassortant forms have caused human infections.

In November 2016, Hunan and Guangxi provinces reported two additional H5N6 human cases, one from each province. Both human cases were characterized by severe pneumonia and resulted in fatal outcomes. Epidemiologic investigations revealed that both patients had a history of exposure to dead chickens. The Chinese National Influenza Center isolated H5N6 viruses in a biosafety level 3 facility by inoculating 0.2 mL of each original sample into 9- to 11-day-old specific pathogen-free (SPF) embryonated chicken egg allantoic cavities. The isolated viruses were termed A/Hunan/55555/2016 (H5N6, HN555) and A/Guangxi/55726/2016 (H5N6, GX726).

The eight gene segments of these two viruses were fully sequenced using an Ion Torrent PGM platform (ThermoFisher, Waltham, MA, USA). Sequences of HN555 or GX726 viruses have been submitted to the Global Initiative on Sharing Avian Influenza Data (GISAID) with the accession numbers EPI873661–EPI873676.

To determine the evolutionary relationship of these two H5N6 viruses, gene sequences were compared with those of other influenza viruses of human or avian origin on GISAID. Phylogenetic analyses of the hemagglutinin (HA) genes showed that both HN555 and GX726 viruses belonged to clade 2.3.4.4 II (Supplementary Figure S1A). Neuraminidase (NA) genes of both H5N6 viruses had an 11-amino acid deletion (residues 59–69) in the stalk region and were grouped into subgroup II (Supplementary Figure S1B). The six internal genes of both H5N6 viruses were classified into several subgroups (Figure 1 and Supplementary Figures S1C–S1H). Except for the PA gene, which clustered into the Eurasian gene pool, the evolutionary lineage of the other internal gene segments of the HN555 virus closely matched the prototype of previously reported H5N6 viruses.^4^ All internal genes of the GX726 virus clustered with the A/Environment/Guangxi/44389/2015 (H5N6) virus (Supplementary Figure S1), which represents one of the previously identified H5N6 genotypes.^4^ These results suggested that the HN555 virus contained a distinctive internal gene cassette and might therefore be a novel H5N6 genotype virus. Although GX726-like genotype viruses have been reported in avian species, the GX726 virus is the first to be isolated from humans.

Basic Local Alignment Search Tool (BLAST) results of the viral nucleotide sequences found that most genes of the GX726 virus had the highest similarity with previously isolated H5N6 viruses. However, the PB1 and PA genes of the HN555 virus exhibited the highest nucleotide sequence homology with the A/duck/Guangxi/175D12/2014 (H3N6, DkD12) and A/duck/Zhejiang/6D7/2013 (H3N2, Dk6D7) isolates, respectively (Supplementary Table S1). These results further indicated that the HN555 virus might be generated by reassortment between H5N6 viruses and DkD12-like/Dk6D7-like viruses.

We next evaluated whether these two novel genotype H5N6 viruses had gained any key mammalian-adapted substitutions. For the HA gene, both viruses had a multiple basic amino acid motif (RERRRKR↓G) at the cleavage sites, indicating their high pathogenicity in poultry. Substitution of Q226L or G228S (H3 numbering) was not detected in either virus, which indicated their preference for avian receptors.^5^ The amino acid 627E in the PB2 protein suggested a possible low viral pathogenicity in mice.^6^ No substitutions associated with drug resistance in the NA or M2 proteins were detected, indicating their sensitivity to neuraminidase inhibitors or adamantine.^7^

In summary, two additional human infections with highly pathogenic influenza A (H5N6) viruses were reported in November 2016 in mainland China. Both homology analysis of nucleotide sequences and phylogenetic analysis of two isolated H5N6 viruses showed that the HN555 virus represented a novel H5N6 genotype and may have been generated by the reassortment of circulating H5N6 viruses with other low-pathogenicity H3 viruses (Figure 1). GX726 represented another novel H5N6 genotype member that has been transmitted from avian species to humans. Our findings suggest that the currently circulating H5N6 viruses in birds may continuously reassort with other enzootic low-pathogenicity influenza viruses. These will likely not be the last novel H5N6 genotype viruses to be generated. Therefore, enhanced surveillance of influenza viruses in birds and humans is continuously needed.

## Figures and Tables

**Figure 1:**
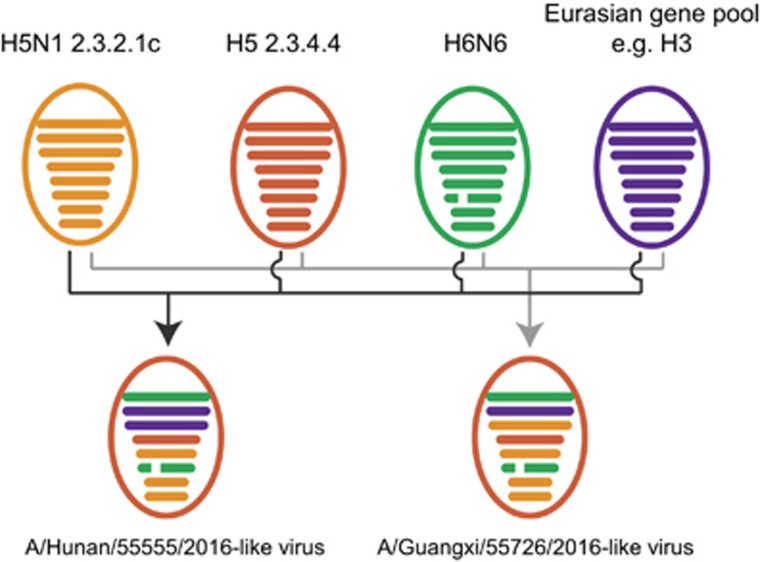
Possible reassortment patterns of A/Hunan/55555/2016 (H5N6, HN555) and A/Guangxi/55726/2016 (H5N6, GX726) viruses. Colored ovals represent influenza virus particles. Eight horizontal bars in ovals represent the gene segments PB2, PB1, PA, HA, NP, NA, M and NS. Segments in descendant viruses are colored according to their corresponding source virus to illustrate gene ancestry through reassortment events. A broken bar in segment 6 (NA) indicates a stalk region deletion.
