# Supplementary material for: Human infections with novel reassortant H5N6 avian influenza viruses in China
Source: Emerg Microbes Infect. 2017 Jun 7;6(6):e50–. doi: 10.1038/emi.2017.38 (PMC5520314; doi:10.1038/emi.2017.38)
Supplement: Supplementary Table S1 [file emi201738x1.docx]

| Virus | Segment | BLAST highest similar virus strain | Identity |
| --- | --- | --- | --- |
| A/Hunan/55555/2016 (H5N6)  A/Hunan/55555/2016 (H5N6)  A/Hunan/55555/2016 (H5N6)  A/Hunan/55555/2016 (H5N6)  A/Hunan/55555/2016 (H5N6)  A/Hunan/55555/2016 (H5N6)  A/Hunan/55555/2016 (H5N6)  A/Hunan/55555/2016 (H5N6) | PB2 | A/Environment/Guangdong/33311/2015(H5N6) | 2246/2280(98.5%) |
|  | PB1 | A/duck/Guangxi/175D12/2014(H3N6) | 2231/2274(98.1%) |
|  | PA | A/duck/Zhejiang/6D7/2013(H3N2) | 2104/2151(97.8%) |
|  | HA | A/Environment/Guangxi/44389/2015(H5N6) | 1684/1707(98.7%) |
|  | NP | A/Syrrhaptes paradoxus/Guangdong/ZH283/2015(H5N6) | 1482/1497(99.0%) |
|  | NA | A/duck/Guangdong/01.01 SZSGXJK007-G/2016(H5N6) | 1365/1380(98.9%) |
|  | MP | A/goose/Guangdong/04.22 DGCP117-O/2015(H5N6) | 976/982(99.4%) |
|  | NS | A/Environment/Guangdong/33311/2015(H5N6) | 816/824(99.0%) |
|  |  |  |  |
| A/Guangxi/55726/2016 (H5N6)  A/Guangxi/55726/2016 (H5N6)  A/Guangxi/55726/2016 (H5N6)  A/Guangxi/55726/2016 (H5N6)  A/Guangxi/55726/2016 (H5N6)  A/Guangxi/55726/2016 (H5N6)  A/Guangxi/55726/2016 (H5N6)  A/Guangxi/55726/2016 (H5N6) | PB2 | A/feline/Guangdong/2/2015(H5N6) | 2247/2280(98.6%) |
|  | PB1 | A/Environment/Guangxi/42586/2015(H5N6) | 2256/2274(99.2%) |
|  | PA | A/Environment/Guangxi/42586/2015(H5N6) | 2139/2151(99.4%) |
|  | HA | A/Environment/Guangxi/44389/2015(H5N6) | 1687/1707(98.8%) |
|  | NP | A/duck/Guangzhou/41227/2014(H5N6) | 1487/1497(99.3%) |
|  | NA | A/duck/Guangdong/04.22 DGCPLB020-O/2015(H5N6) | 1351/1380(97.9%) |
|  | MP | A/Environment/Guangdong/40929/2015(H5N6) | 981/982(99.9%) |
|  | NS | A/duck/Guangzhou/018/2014(H5N6) | 816/823(99.1%) |

**Supplementary Table S1 Genetic compositions of A/Hunan/55555/2016 (H5N6) and A/Guangxi/55726/2016 (H5N6) viruses**
