# Supplementary material for: Human infections with novel reassortant H5N6 avian influenza viruses in China
Source: Emerg Microbes Infect. 2017 Jun 7;6(6):e50–. doi: 10.1038/emi.2017.38 (PMC5520314; doi:10.1038/emi.2017.38)
Supplement: Supplementary Figure [file emi201738x2.pdf]

Supplementary Figure S1 Phylogenetic trees of full-length HA (A), NA (B), PB2 (C), PB1 (D), PA (E), NP (F), M (G), and NS (H) genes of H5N6 influenza A viruses

Human origin H5N6 viruses which were identified in this study (A/Hunan/55555/2016 (H5N6) and A/Guangxi/55726/2016 (H5N6)) or previous studies were highlighted in red or blue. A/Environment/Guangxi/44389/2015 (H5N6) virus, with all eight genes clustered with those of GX726 virus, was highlighted in purple.

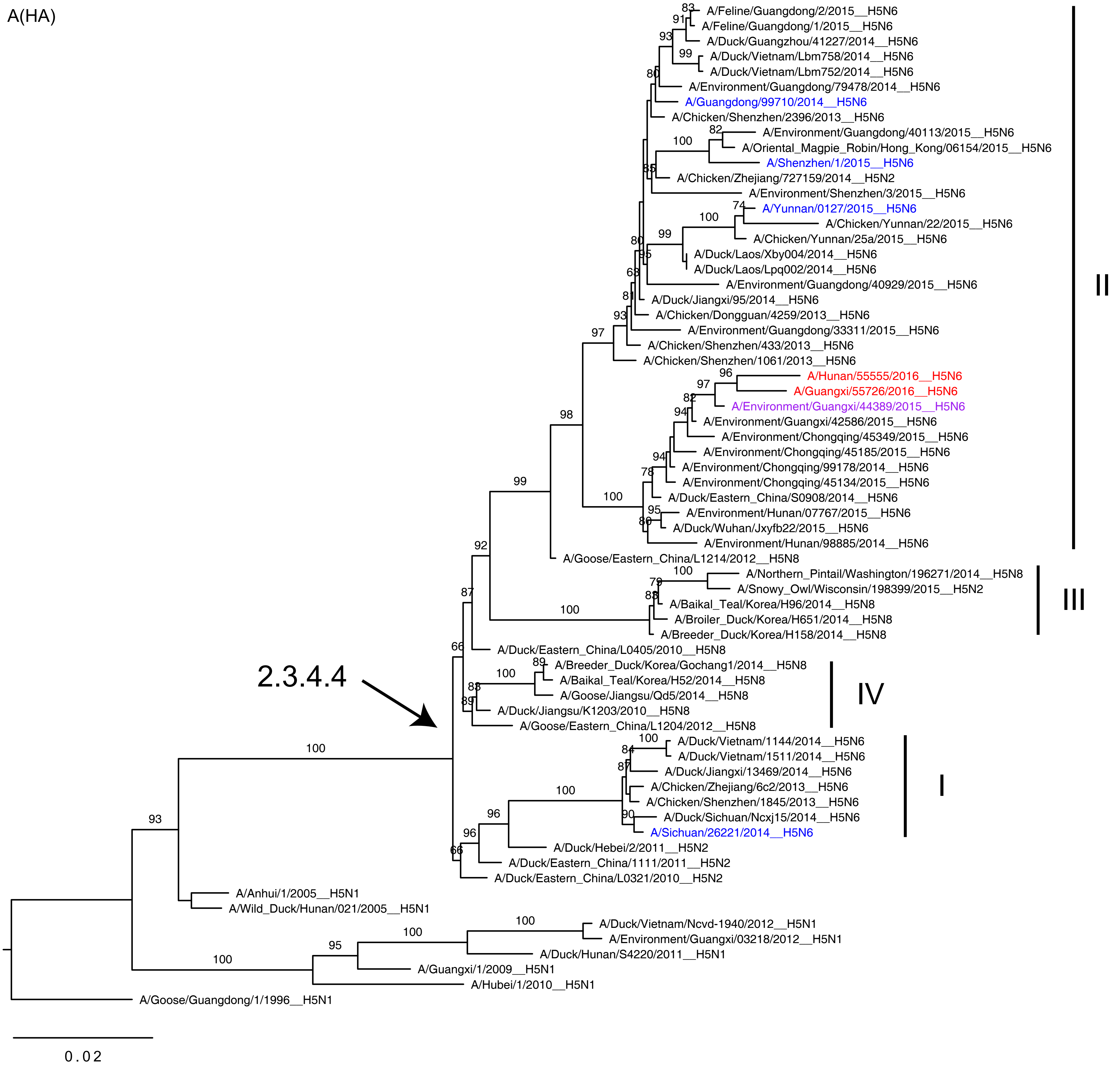
A(HA)
A/Feline/Guangdong/2/2015__H5N6
A/Feline/Guangdong/1/2015__H5N6
A/Duck/Guangzhou/41227/2014__H5N6
A/Duck/Vietnam/Lbm758/2014__H5N6
A/Duck/Vietnam/Lbm752/2014__H5N6
A/Environment/Guangdong/79478/2014__H5N6
A/Guangdong/99710/2014__H5N6
A/Chicken/Shenzhen/2396/2013__H5N6
A/Environment/Guangdong/40113/2015__H5N6
A/Oriental_Magpie_Robin/Hong_Kong/06154/2015__H5N6
A/Shenzhen/1/2015__H5N6
A/Chicken/Zhejiang/727159/2014__H5N2
A/Environment/Shenzhen/3/2015__H5N6
A/Yunnan/0127/2015__H5N6
A/Chicken/Yunnan/22/2015__H5N6
A/Chicken/Yunnan/25a/2015__H5N6
A/Duck/Laos/Xby004/2014__H5N6
A/Duck/Laos/Lpq002/2014__H5N6
A/Environment/Guangdong/40929/2015__H5N6
A/Duck/Jiangxi/95/2014__H5N6
A/Chicken/Dongguan/4259/2013__H5N6
A/Environment/Guangdong/33311/2015__H5N6
A/Chicken/Shenzhen/433/2013__H5N6
A/Chicken/Shenzhen/1061/2013__H5N6
A/Hunan/55555/2016__H5N6
A/Guangxi/55726/2016__H5N6
A/Environment/Guangxi/44389/2015__H5N6
A/Environment/Guangxi/42586/2015__H5N6
A/Environment/Chongqing/45349/2015__H5N6
A/Environment/Chongqing/45185/2015__H5N6
A/Environment/Chongqing/99178/2014__H5N6
A/Environment/Chongqing/45134/2015__H5N6
A/Duck/Eastern_China/S0908/2014__H5N6
A/Environment/Hunan/07767/2015__H5N6
A/Duck/Wuhan/Jxyfb22/2015__H5N6
A/Environment/Hunan/98885/2014__H5N6
A/Goose/Eastern_China/L1214/2012__H5N8
II
A/Northern_Pintail/Washington/196271/2014__H5N8
A/Snowy_Owl/Wisconsin/198399/2015__H5N2
A/Baikal_Teal/Korea/H96/2014__H5N8
A/Broiler_Duck/Korea/H651/2014__H5N8
A/Breeder_Duck/Korea/H158/2014__H5N8
III
A/Duck/Eastern_China/L0405/2010__H5N8
A/Breeder_Duck/Korea/Gochang1/2014__H5N8
A/Baikal_Teal/Korea/H52/2014__H5N8
A/Goose/Jiangsu/Qd5/2014__H5N8
A/Duck/Jiangsu/K1203/2010__H5N8
A/Goose/Eastern_China/L1204/2012__H5N8
IV
2.3.4.4
A/Duck/Vietnam/1144/2014__H5N6
A/Duck/Vietnam/1511/2014__H5N6
A/Duck/Jiangxi/13469/2014__H5N6
A/Chicken/Zhejiang/6c2/2013__H5N6
A/Chicken/Shenzhen/1845/2013__H5N6
A/Duck/Sichuan/Ncxj15/2014__H5N6
A/Sichuan/26221/2014__H5N6
I
A/Duck/Hebei/2/2011__H5N2
A/Duck/Eastern_China/1111/2011__H5N2
A/Duck/Eastern_China/L0321/2010__H5N2
A/Anhui/1/2005__H5N1
A/Wild_Duck/Hunan/021/2005__H5N1
A/Duck/Vietnam/Ncvd-1940/2012__H5N1
A/Environment/Guangxi/03218/2012__H5N1
A/Duck/Hunan/S4220/2011__H5N1
A/Guangxi/1/2009__H5N1
A/Hubei/1/2010__H5N1
A/Goose/Guangdong/1/1996__H5N1
0.02

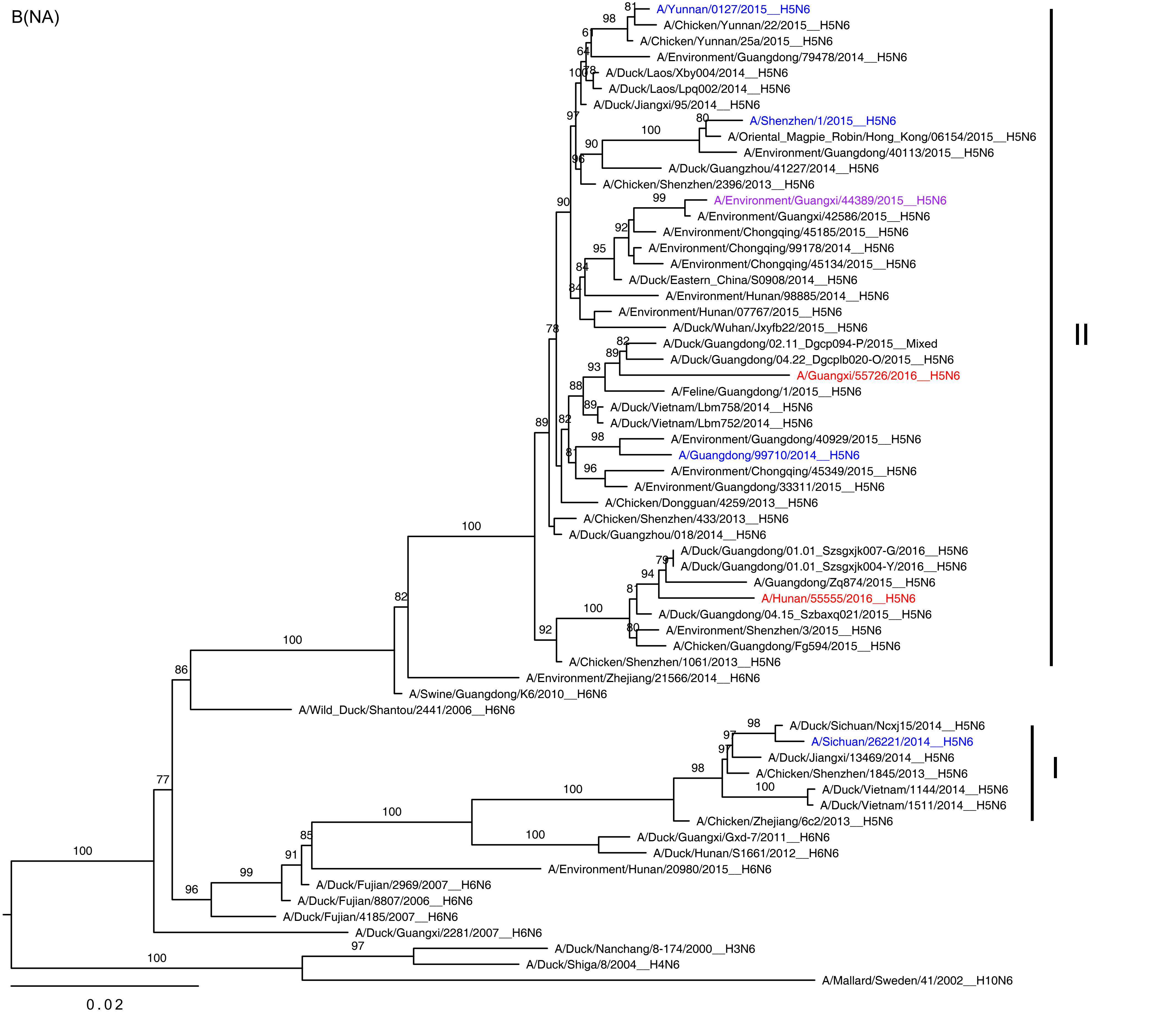

B(NA)
A/Yunnan/0127/2015__H5N6
A/Chicken/Yunnan/22/2015__H5N6
A/Chicken/Yunnan/25a/2015__H5N6
A/Environment/Guangdong/79478/2014__H5N6
A/Duck/Laos/Xby004/2014__H5N6
A/Duck/Laos/Lpq002/2014__H5N6
A/Duck/Jiangxi/95/2014__H5N6
A/Shenzhen/1/2015__H5N6
A/Oriental_Magpie_Robin/Hong_Kong/06154/2015__H5N6
A/Environment/Guangdong/40113/2015__H5N6
A/Duck/Guangzhou/41227/2014__H5N6
A/Chicken/Shenzhen/2396/2013__H5N6
A/Environment/Guangxi/44389/2015__H5N6
A/Environment/Guangxi/42586/2015__H5N6
A/Environment/Chongqing/45185/2015__H5N6
A/Environment/Chongqing/99178/2014__H5N6
A/Environment/Chongqing/45134/2015__H5N6
A/Duck/Eastern_China/S0908/2014__H5N6
A/Environment/Hunan/98885/2014__H5N6
A/Environment/Hunan/07767/2015__H5N6
A/Duck/Wuhan/Jxyfb22/2015__H5N6
A/Duck/Guangdong/02.11_Dgcp094-P/2015__Mixed
A/Duck/Guangdong/04.22_Dgcplb020-O/2015__H5N6
A/Guangxi/55726/2016__H5N6
A/Feline/Guangdong/1/2015__H5N6
A/Duck/Vietnam/Lbm758/2014__H5N6
A/Duck/Vietnam/Lbm752/2014__H5N6
A/Environment/Guangdong/40929/2015__H5N6
A/Guangdong/99710/2014__H5N6
A/Environment/Chongqing/45349/2015__H5N6
A/Environment/Guangdong/33311/2015__H5N6
A/Chicken/Dongguan/4259/2013__H5N6
A/Chicken/Shenzhen/433/2013__H5N6
A/Duck/Guangzhou/018/2014__H5N6
A/Duck/Guangdong/01.01_Szsgxjk007-G/2016__H5N6
A/Duck/Guangdong/01.01_Szsgxjk004-Y/2016__H5N6
A/Guangdong/Zq874/2015__H5N6
A/Hunan/55555/2016__H5N6
A/Duck/Guangdong/04.15_Szbaxq021/2015__H5N6
A/Environment/Shenzhen/3/2015__H5N6
A/Chicken/Guangdong/Fg594/2015__H5N6
A/Chicken/Shenzhen/1061/2013__H5N6
A/Environment/Zhejiang/21566/2014__H6N6
A/Swine/Guangdong/K6/2010__H6N6
A/Wild_Duck/Shantou/2441/2006__H6N6
A/Duck/Sichuan/Ncxj15/2014__H5N6
A/Sichuan/26221/2014__H5N6
A/Duck/Jiangxi/13469/2014__H5N6
A/Chicken/Shenzhen/1845/2013__H5N6
A/Duck/Vietnam/1144/2014__H5N6
A/Duck/Vietnam/1511/2014__H5N6
A/Chicken/Zhejiang/6c2/2013__H5N6
A/Duck/Guangxi/Gxd-7/2011__H6N6
A/Duck/Hunan/S1661/2012__H6N6
A/Environment/Hunan/20980/2015__H6N6
A/Duck/Fujian/2969/2007__H6N6
A/Duck/Fujian/8807/2006__H6N6
A/Duck/Fujian/4185/2007__H6N6
A/Duck/Guangxi/2281/2007__H6N6
A/Duck/Nanchang/8-174/2000__H3N6
A/Duck/Shiga/8/2004__H4N6
A/Mallard/Sweden/41/2002__H10N6
II
I
0.02

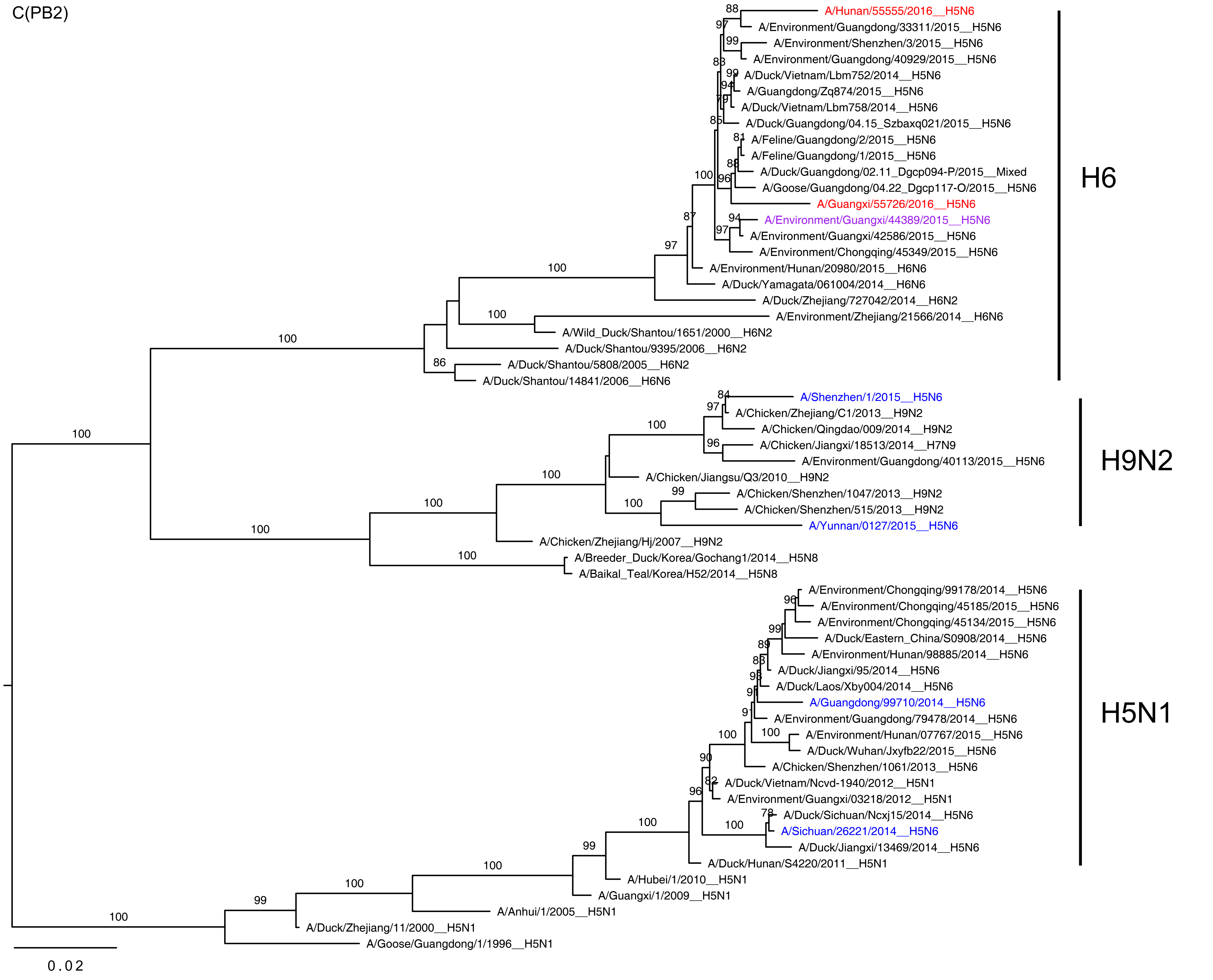
C(PB2)
A/Hunan/55555/2016__H5N6
A/Environment/Guangdong/33311/2015__H5N6
A/Environment/Shenzhen/3/2015__H5N6
A/Environment/Guangdong/40929/2015__H5N6
A/Duck/Vietnam/Lbm752/2014__H5N6
A/Guangdong/Zq874/2015__H5N6
A/Duck/Vietnam/Lbm758/2014__H5N6
A/Duck/Guangdong/04.15_Szbaxq021/2015__H5N6
A/Feline/Guangdong/2/2015__H5N6
A/Feline/Guangdong/1/2015__H5N6
A/Duck/Guangdong/02.11_Dgcp094-P/2015__Mixed
A/Goose/Guangdong/04.22_Dgcp117-O/2015__H5N6
A/Guangxi/55726/2016__H5N6
A/Environment/Guangxi/44389/2015__H5N6
A/Environment/Guangxi/42586/2015__H5N6
A/Environment/Chongqing/45349/2015__H5N6
A/Environment/Hunan/20980/2015__H6N6
A/Duck/Yamagata/061004/2014__H6N6
A/Duck/Zhejiang/727042/2014__H6N2
A/Environment/Zhejiang/21566/2014__H6N6
A/Wild_Duck/Shantou/1651/2000__H6N2
A/Duck/Shantou/9395/2006__H6N2
A/Duck/Shantou/5808/2005__H6N2
A/Duck/Shantou/14841/2006__H6N6
H6
A/Shenzhen/1/2015__H5N6
A/Chicken/Zhejiang/C1/2013__H9N2
A/Chicken/Qingdao/009/2014__H9N2
A/Chicken/Jiangxi/18513/2014__H7N9
A/Environment/Guangdong/40113/2015__H5N6
A/Chicken/Jiangsu/Q3/2010__H9N2
A/Chicken/Shenzhen/1047/2013__H9N2
A/Chicken/Shenzhen/515/2013__H9N2
A/Yunnan/0127/2015__H5N6
A/Chicken/Zhejiang/Hj/2007__H9N2
A/Breeder_Duck/Korea/Gochang1/2014__H5N8
A/Baikal_Teal/Korea/H52/2014__H5N8
H9N2
A/Environment/Chongqing/99178/2014__H5N6
A/Environment/Chongqing/45185/2015__H5N6
A/Environment/Chongqing/45134/2015__H5N6
A/Duck/Eastern_China/S0908/2014__H5N6
A/Environment/Hunan/98885/2014__H5N6
A/Duck/Jiangxi/95/2014__H5N6
A/Duck/Laos/Xby004/2014__H5N6
A/Guangdong/99710/2014__H5N6
A/Environment/Guangdong/79478/2014__H5N6
A/Environment/Hunan/07767/2015__H5N6
A/Duck/Wuhan/Jxyfb22/2015__H5N6
A/Chicken/Shenzhen/1061/2013__H5N6
A/Duck/Vietnam/Ncvd-1940/2012__H5N1
A/Environment/Guangxi/03218/2012__H5N1
A/Duck/Sichuan/Ncxj15/2014__H5N6
A/Sichuan/26221/2014__H5N6
A/Duck/Jiangxi/13469/2014__H5N6
A/Duck/Hunan/S4220/2011__H5N1
A/Hubei/1/2010__H5N1
A/Guangxi/1/2009__H5N1
A/Anhui/1/2005__H5N1
A/Duck/Zhejiang/11/2000__H5N1
A/Goose/Guangdong/1/1996__H5N1
H5N1
0.02

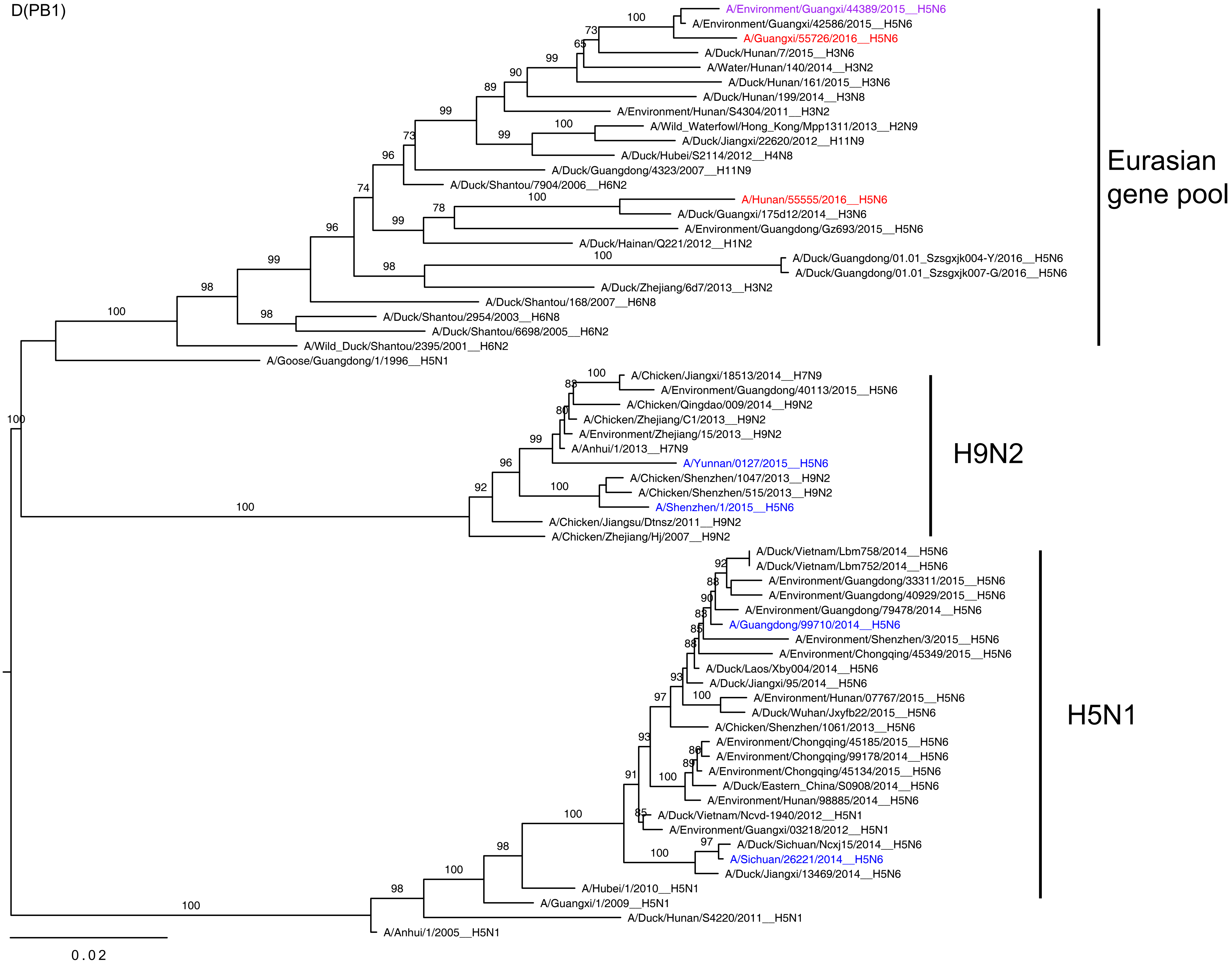
D(PB1)
A/Environment/Guangxi/44389/2015__H5N6
A/Environment/Guangxi/42586/2015__H5N6
A/Guangxi/55726/2016__H5N6
A/Duck/Hunan/7/2015__H3N6
A/Water/Hunan/140/2014__H3N2
A/Duck/Hunan/161/2015__H3N6
A/Duck/Hunan/199/2014__H3N8
A/Environment/Hunan/S4304/2011__H3N2
A/Wild_Waterfowl/Hong_Kong/Mpp1311/2013__H2N9
A/Duck/Jiangxi/22620/2012__H11N9
A/Duck/Hubei/S2114/2012__H4N8
A/Duck/Guangdong/4323/2007__H11N9
A/Duck/Shantou/7904/2006__H6N2
A/Hunan/55555/2016__H5N6
A/Duck/Guangxi/175d12/2014__H3N6
A/Environment/Guangdong/Gz693/2015__H5N6
A/Duck/Hainan/Q221/2012__H1N2
A/Duck/Guangdong/01.01_Szsgxjk004-Y/2016__H5N6
A/Duck/Guangdong/01.01_Szsgxjk007-G/2016__H5N6
A/Duck/Zhejiang/6d7/2013__H3N2
A/Duck/Shantou/168/2007__H6N8
A/Duck/Shantou/2954/2003__H6N8
A/Duck/Shantou/6698/2005__H6N2
A/Wild_Duck/Shantou/2395/2001__H6N2
A/Goose/Guangdong/1/1996__H5N1
Eurasian gene pool
A/Chicken/Jiangxi/18513/2014__H7N9
A/Environment/Guangdong/40113/2015__H5N6
A/Chicken/Qingdao/009/2014__H9N2
A/Chicken/Zhejiang/C1/2013__H9N2
A/Environment/Zhejiang/15/2013__H9N2
A/Anhui/1/2013__H7N9
A/Yunnan/0127/2015__H5N6
A/Chicken/Shenzhen/1047/2013__H9N2
A/Chicken/Shenzhen/515/2013__H9N2
A/Shenzhen/1/2015__H5N6
A/Chicken/Jiangsu/Dtnsz/2011__H9N2
A/Chicken/Zhejiang/Hj/2007__H9N2
H9N2
A/Duck/Vietnam/Lbm758/2014__H5N6
A/Duck/Vietnam/Lbm752/2014__H5N6
A/Environment/Guangdong/33311/2015__H5N6
A/Environment/Guangdong/40929/2015__H5N6
A/Environment/Guangdong/79478/2014__H5N6
A/Guangdong/99710/2014__H5N6
A/Environment/Shenzhen/3/2015__H5N6
A/Environment/Chongqing/45349/2015__H5N6
A/Duck/Laos/Xby004/2014__H5N6
A/Duck/Jiangxi/95/2014__H5N6
A/Environment/Hunan/07767/2015__H5N6
A/Duck/Wuhan/Jxyfb22/2015__H5N6
A/Chicken/Shenzhen/1061/2013__H5N6
A/Environment/Chongqing/45185/2015__H5N6
A/Environment/Chongqing/99178/2014__H5N6
A/Environment/Chongqing/45134/2015__H5N6
A/Duck/Eastern_China/S0908/2014__H5N6
A/Environment/Hunan/98885/2014__H5N6
A/Duck/Vietnam/Ncvd-1940/2012__H5N1
A/Environment/Guangxi/03218/2012__H5N1
A/Duck/Sichuan/Ncxj15/2014__H5N6
A/Sichuan/26221/2014__H5N6
A/Duck/Jiangxi/13469/2014__H5N6
A/Hubei/1/2010__H5N1
A/Guangxi/1/2009__H5N1
A/Duck/Hunan/S4220/2011__H5N1
A/Anhui/1/2005__H5N1
H5N1
0.02

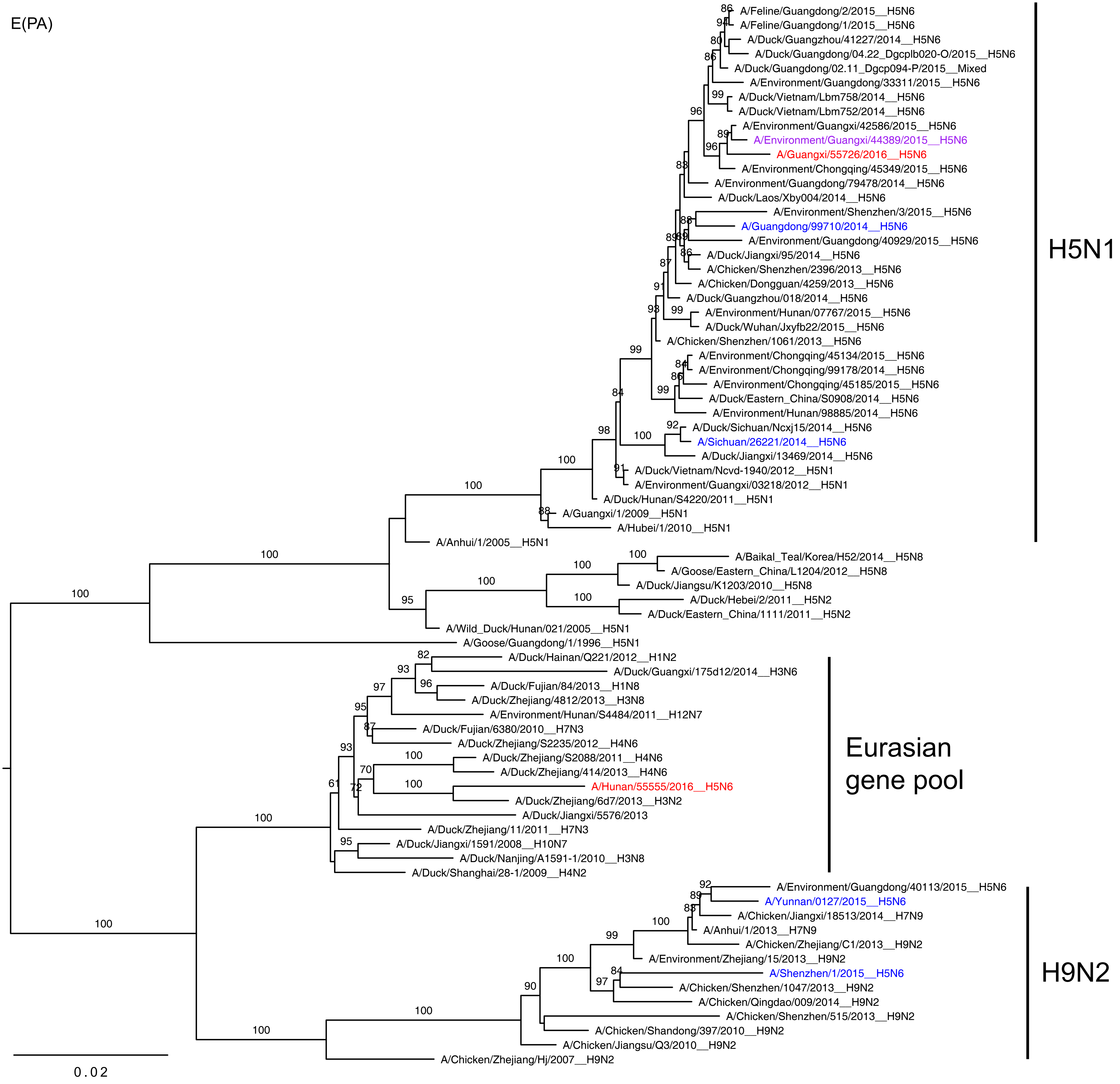

E(PA)
A/Feline/Guangdong/2/2015__H5N6
A/Feline/Guangdong/1/2015__H5N6
A/Duck/Guangzhou/41227/2014__H5N6
A/Duck/Guangdong/04.22_Dgcplb020-O/2015__H5N6
A/Duck/Guangdong/02.11_Dgcp094-P/2015__Mixed
A/Environment/Guangdong/33311/2015__H5N6
A/Duck/Vietnam/Lbm758/2014__H5N6
A/Duck/Vietnam/Lbm752/2014__H5N6
A/Environment/Guangxi/42586/2015__H5N6
A/Environment/Guangxi/44389/2015__H5N6
A/Guangxi/55726/2016__H5N6
A/Environment/Chongqing/45349/2015__H5N6
A/Environment/Guangdong/79478/2014__H5N6
A/Duck/Laos/Xby004/2014__H5N6
A/Environment/Shenzhen/3/2015__H5N6
A/Guangdong/99710/2014__H5N6
A/Environment/Guangdong/40929/2015__H5N6
A/Duck/Jiangxi/95/2014__H5N6
A/Chicken/Shenzhen/2396/2013__H5N6
A/Chicken/Dongguan/4259/2013__H5N6
A/Duck/Guangzhou/018/2014__H5N6
A/Environment/Hunan/07767/2015__H5N6
A/Duck/Wuhan/Jxyfb22/2015__H5N6
A/Chicken/Shenzhen/1061/2013__H5N6
A/Environment/Chongqing/45134/2015__H5N6
A/Environment/Chongqing/99178/2014__H5N6
A/Environment/Chongqing/45185/2015__H5N6
A/Duck/Eastern_China/S0908/2014__H5N6
A/Environment/Hunan/98885/2014__H5N6
A/Duck/Sichuan/Ncxj15/2014__H5N6
A/Sichuan/26221/2014__H5N6
A/Duck/Jiangxi/13469/2014__H5N6
A/Duck/Vietnam/Ncvd-1940/2012__H5N1
A/Environment/Guangxi/03218/2012__H5N1
A/Duck/Hunan/S4220/2011__H5N1
A/Guangxi/1/2009__H5N1
A/Hubei/1/2010__H5N1
A/Anhui/1/2005__H5N1
A/Baikal_Teal/Korea/H52/2014__H5N8
A/Goose/Eastern_China/L1204/2012__H5N8
A/Duck/Jiangsu/K1203/2010__H5N8
A/Duck/Hebei/2/2011__H5N2
A/Duck/Eastern_China/1111/2011__H5N2
A/Wild_Duck/Hunan/021/2005__H5N1
A/Goose/Guangdong/1/1996__H5N1
H5N1
A/Duck/Hainan/Q221/2012__H1N2
A/Duck/Guangxi/175d12/2014__H3N6
A/Duck/Fujian/84/2013__H1N8
A/Duck/Zhejiang/4812/2013__H3N8
A/Environment/Hunan/S4484/2011__H12N7
A/Duck/Fujian/6380/2010__H7N3
A/Duck/Zhejiang/S2235/2012__H4N6
A/Duck/Zhejiang/S2088/2011__H4N6
A/Duck/Zhejiang/414/2013__H4N6
A/Hunan/55555/2016__H5N6
A/Duck/Zhejiang/6d7/2013__H3N2
A/Duck/Jiangxi/5576/2013
A/Duck/Zhejiang/11/2011__H7N3
A/Duck/Jiangxi/1591/2008__H10N7
A/Duck/Nanjing/A1591-1/2010__H3N8
A/Duck/Shanghai/28-1/2009__H4N2
Eurasian gene pool
A/Environment/Guangdong/40113/2015__H5N6
A/Yunnan/0127/2015__H5N6
A/Chicken/Jiangxi/18513/2014__H7N9
A/Anhui/1/2013__H7N9
A/Chicken/Zhejiang/C1/2013__H9N2
A/Environment/Zhejiang/15/2013__H9N2
A/Shenzhen/1/2015__H5N6
A/Chicken/Shenzhen/1047/2013__H9N2
A/Chicken/Qingdao/009/2014__H9N2
A/Chicken/Shenzhen/515/2013__H9N2
A/Chicken/Shandong/397/2010__H9N2
A/Chicken/Jiangsu/Q3/2010__H9N2
A/Chicken/Zhejiang/Hj/2007__H9N2
H9N2
0.02

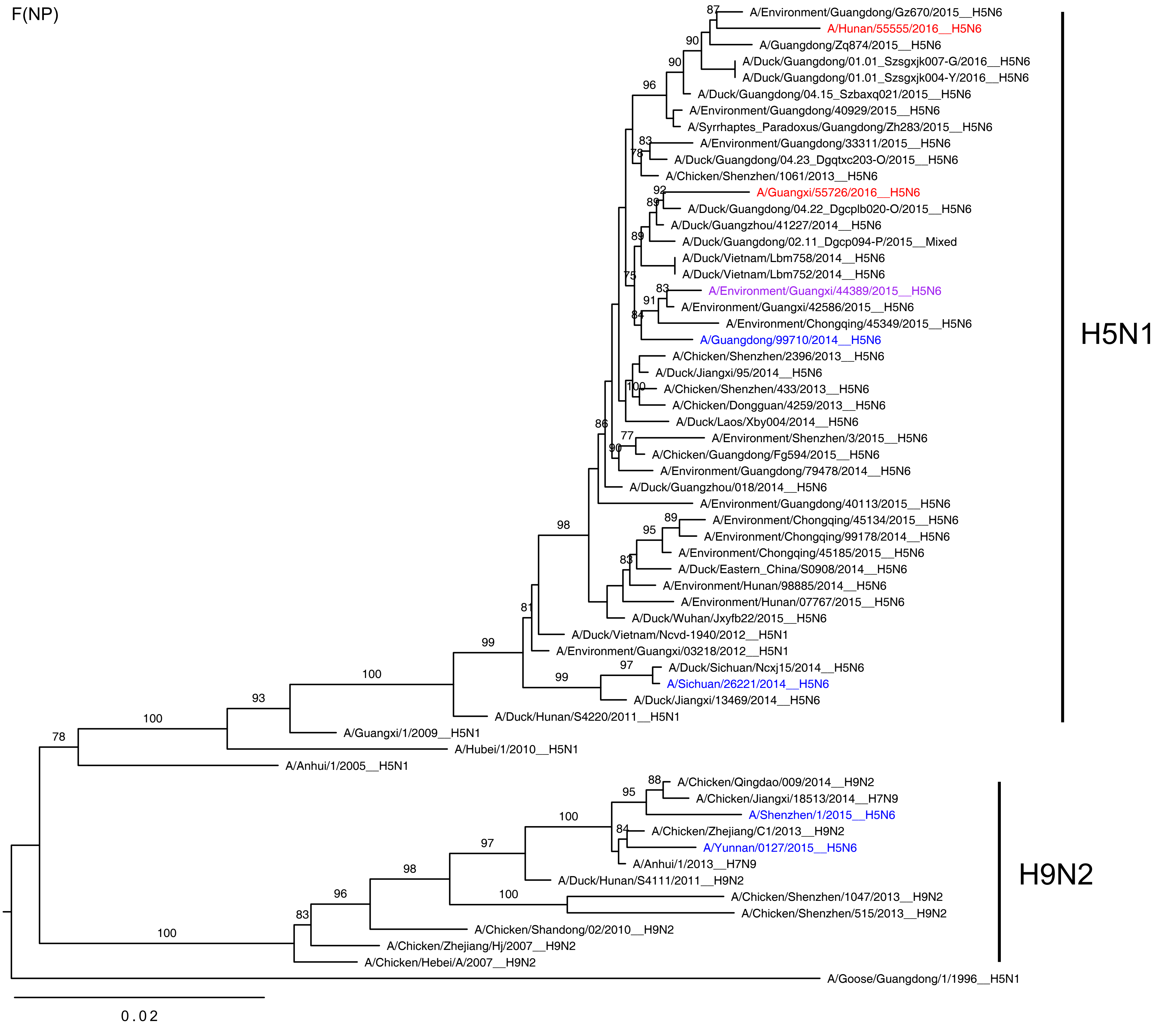

F(NP)
H5N1
H9N2
A/Environment/Guangdong/Gz670/2015__H5N6
A/Hunan/55555/2016__H5N6
A/Guangdong/Zq874/2015__H5N6
A/Duck/Guangdong/01.01_Szsgxjk007-G/2016__H5N6
A/Duck/Guangdong/01.01_Szsgxjk004-Y/2016__H5N6
A/Duck/Guangdong/04.15_Szbaxq021/2015__H5N6
A/Environment/Guangdong/40929/2015__H5N6
A/Syrrhaptes_Paradoxus/Guangdong/Zh283/2015__H5N6
A/Environment/Guangdong/33311/2015__H5N6
A/Duck/Guangdong/04.23_Dgqtxc203-O/2015__H5N6
A/Chicken/Shenzhen/1061/2013__H5N6
A/Guangxi/55726/2016__H5N6
A/Duck/Guangdong/04.22_Dgcplb020-O/2015__H5N6
A/Duck/Guangzhou/41227/2014__H5N6
A/Duck/Guangdong/02.11_Dgcp094-P/2015__Mixed
A/Duck/Vietnam/Lbm758/2014__H5N6
A/Duck/Vietnam/Lbm752/2014__H5N6
A/Environment/Guangxi/44389/2015__H5N6
A/Environment/Guangxi/42586/2015__H5N6
A/Environment/Chongqing/45349/2015__H5N6
A/Guangdong/99710/2014__H5N6
A/Chicken/Shenzhen/2396/2013__H5N6
A/Duck/Jiangxi/95/2014__H5N6
A/Chicken/Shenzhen/433/2013__H5N6
A/Chicken/Dongguan/4259/2013__H5N6
A/Duck/Laos/Xby004/2014__H5N6
A/Environment/Shenzhen/3/2015__H5N6
A/Chicken/Guangdong/Fg594/2015__H5N6
A/Environment/Guangdong/79478/2014__H5N6
A/Duck/Guangzhou/018/2014__H5N6
A/Environment/Guangdong/40113/2015__H5N6
A/Environment/Chongqing/45134/2015__H5N6
A/Environment/Chongqing/99178/2014__H5N6
A/Environment/Chongqing/45185/2015__H5N6
A/Duck/Eastern_China/S0908/2014__H5N6
A/Environment/Hunan/98885/2014__H5N6
A/Environment/Hunan/07767/2015__H5N6
A/Duck/Wuhan/Jxyfb22/2015__H5N6
A/Duck/Vietnam/Ncvd-1940/2012__H5N1
A/Environment/Guangxi/03218/2012__H5N1
A/Duck/Sichuan/Ncxj15/2014__H5N6
A/Sichuan/26221/2014__H5N6
A/Duck/Jiangxi/13469/2014__H5N6
A/Duck/Hunan/S4220/2011__H5N1
A/Guangxi/1/2009__H5N1
A/Hubei/1/2010__H5N1
A/Anhui/1/2005__H5N1
A/Chicken/Qingdao/009/2014__H9N2
A/Chicken/Jiangxi/18513/2014__H7N9
A/Shenzhen/1/2015__H5N6
A/Chicken/Zhejiang/C1/2013__H9N2
A/Yunnan/0127/2015__H5N6
A/Anhui/1/2013__H7N9
A/Duck/Hunan/S4111/2011__H9N2
A/Chicken/Shenzhen/1047/2013__H9N2
A/Chicken/Shenzhen/515/2013__H9N2
A/Chicken/Shandong/02/2010__H9N2
A/Chicken/Zhejiang/Hj/2007__H9N2
A/Chicken/Hebei/A/2007__H9N2
A/Goose/Guangdong/1/1996__H5N1
0.02

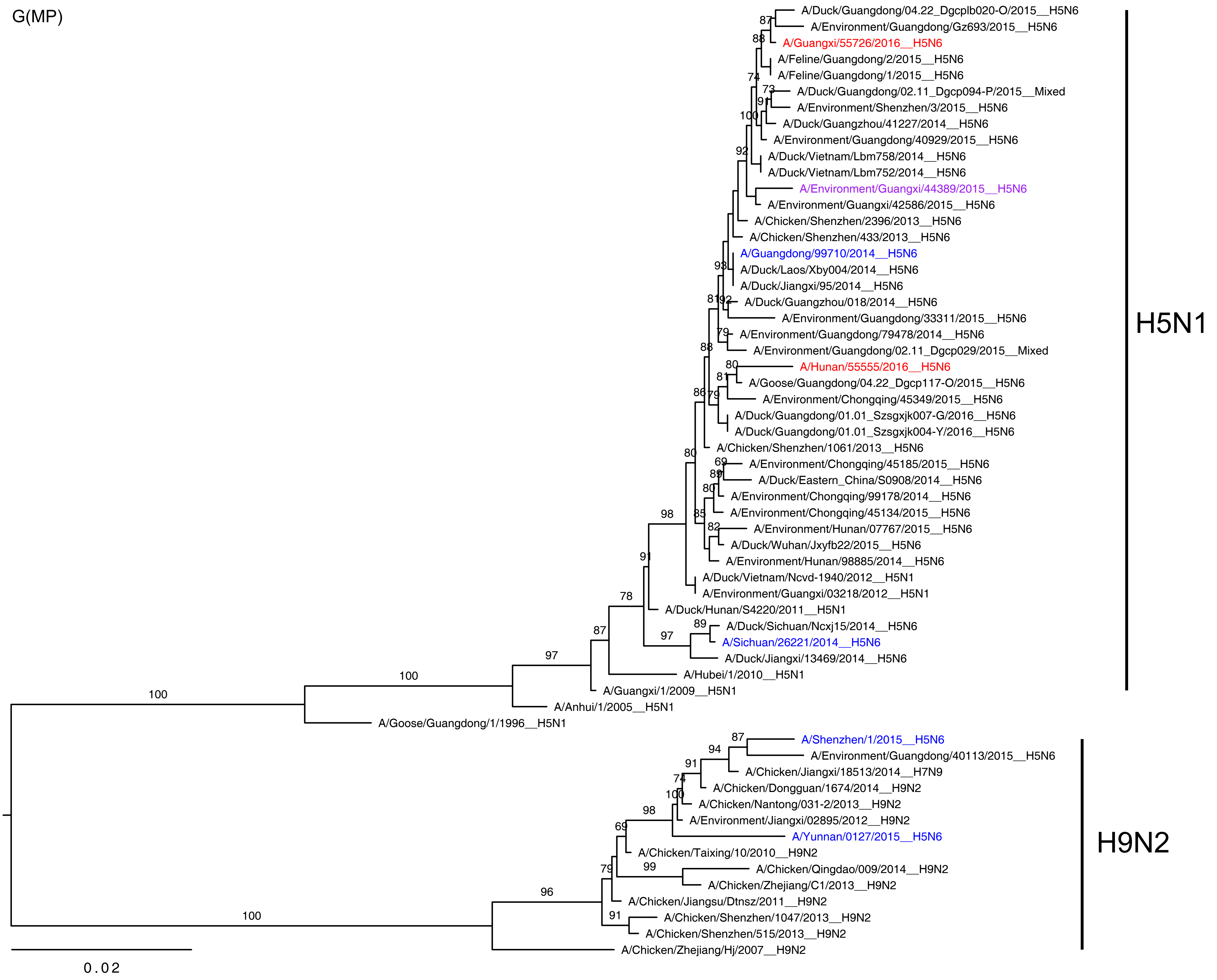

G(MP)
A/Duck/Guangdong/04.22_Dgcplb020-O/2015__H5N6
A/Environment/Guangdong/Gz693/2015__H5N6
A/Guangxi/55726/2016__H5N6
A/Feline/Guangdong/2/2015__H5N6
A/Feline/Guangdong/1/2015__H5N6
A/Duck/Guangdong/02.11_Dgcp094-P/2015__Mixed
A/Environment/Shenzhen/3/2015__H5N6
A/Duck/Guangzhou/41227/2014__H5N6
A/Environment/Guangdong/40929/2015__H5N6
A/Duck/Vietnam/Lbm758/2014__H5N6
A/Duck/Vietnam/Lbm752/2014__H5N6
A/Environment/Guangxi/44389/2015__H5N6
A/Environment/Guangxi/42586/2015__H5N6
A/Chicken/Shenzhen/2396/2013__H5N6
A/Chicken/Shenzhen/433/2013__H5N6
A/Guangdong/99710/2014__H5N6
A/Duck/Laos/Xby004/2014__H5N6
A/Duck/Jiangxi/95/2014__H5N6
A/Duck/Guangzhou/018/2014__H5N6
A/Environment/Guangdong/33311/2015__H5N6
A/Environment/Guangdong/79478/2014__H5N6
A/Environment/Guangdong/02.11_Dgcp029/2015__Mixed
A/Hunan/55555/2016__H5N6
A/Goose/Guangdong/04.22_Dgcp117-O/2015__H5N6
A/Environment/Chongqing/45349/2015__H5N6
A/Duck/Guangdong/01.01_Szsgxjk007-G/2016__H5N6
A/Duck/Guangdong/01.01_Szsgxjk004-Y/2016__H5N6
A/Chicken/Shenzhen/1061/2013__H5N6
A/Environment/Chongqing/45185/2015__H5N6
A/Duck/Eastern_China/S0908/2014__H5N6
A/Environment/Chongqing/99178/2014__H5N6
A/Environment/Chongqing/45134/2015__H5N6
A/Environment/Hunan/07767/2015__H5N6
A/Duck/Wuhan/Jxyfb22/2015__H5N6
A/Environment/Hunan/98885/2014__H5N6
A/Duck/Vietnam/Ncvd-1940/2012__H5N1
A/Environment/Guangxi/03218/2012__H5N1
A/Duck/Hunan/S4220/2011__H5N1
A/Duck/Sichuan/Ncxj15/2014__H5N6
A/Sichuan/26221/2014__H5N6
A/Duck/Jiangxi/13469/2014__H5N6
A/Hubei/1/2010__H5N1
A/Guangxi/1/2009__H5N1
A/Anhui/1/2005__H5N1
A/Goose/Guangdong/1/1996__H5N1
A/Shenzhen/1/2015__H5N6
A/Environment/Guangdong/40113/2015__H5N6
A/Chicken/Jiangxi/18513/2014__H7N9
A/Chicken/Dongguan/1674/2014__H9N2
A/Chicken/Nantong/031-2/2013__H9N2
A/Environment/Jiangxi/02895/2012__H9N2
A/Yunnan/0127/2015__H5N6
A/Chicken/Taixing/10/2010__H9N2
A/Chicken/Qingdao/009/2014__H9N2
A/Chicken/Zhejiang/C1/2013__H9N2
A/Chicken/Jiangsu/Dtnsz/2011__H9N2
A/Chicken/Shenzhen/1047/2013__H9N2
A/Chicken/Shenzhen/515/2013__H9N2
A/Chicken/Zhejiang/Hj/2007__H9N2
H5N1
H9N2
0.02

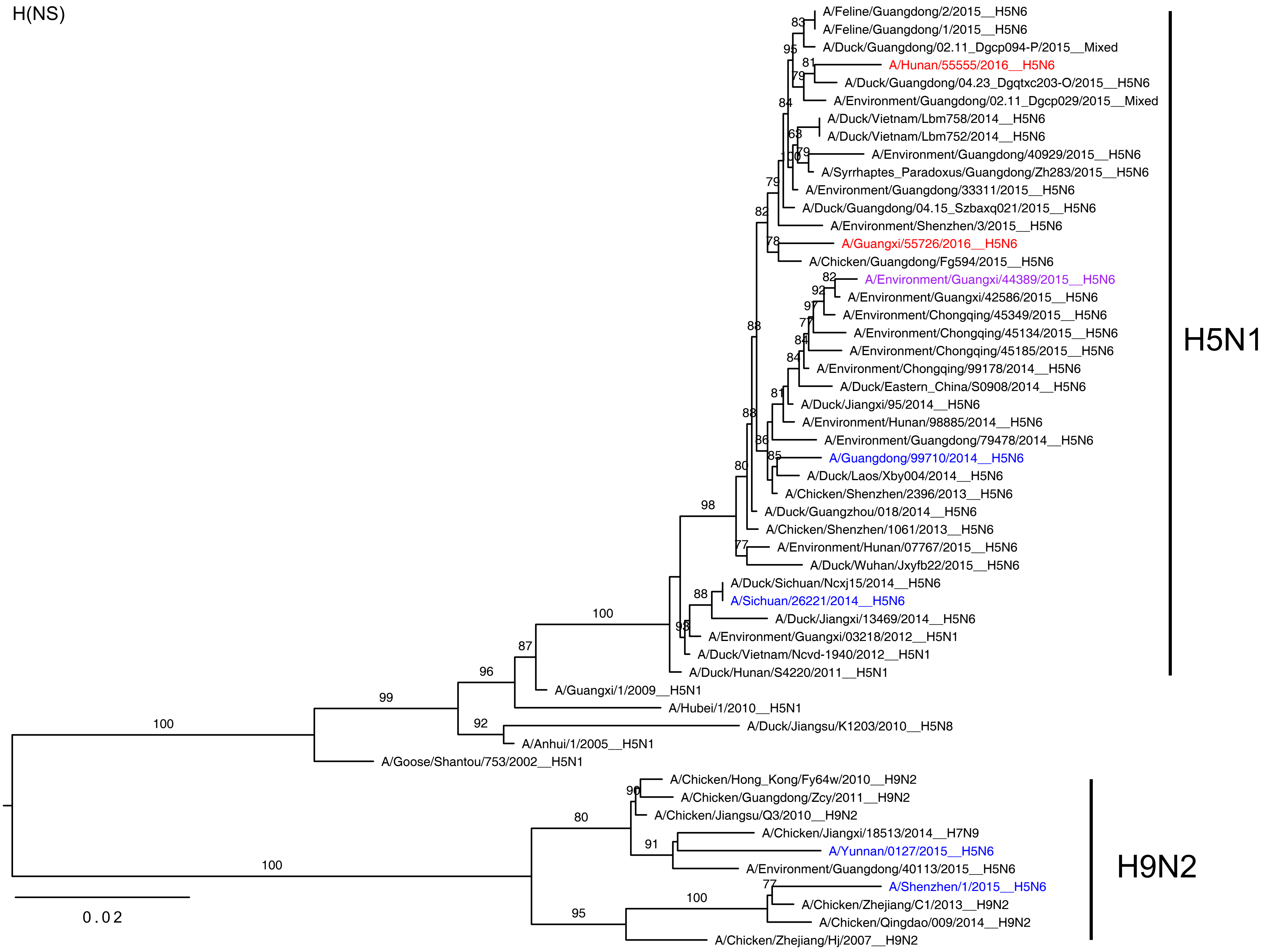
H(NS)
A/Feline/Guangdong/2/2015__H5N6
A/Feline/Guangdong/1/2015__H5N6
A/Duck/Guangdong/02.11_Dgcp094-P/2015__Mixed
A/Hunan/55555/2016__H5N6
A/Duck/Guangdong/04.23_Dgqtxc203-O/2015__H5N6
A/Environment/Guangdong/02.11_Dgcp029/2015__Mixed
A/Duck/Vietnam/Lbm758/2014__H5N6
A/Duck/Vietnam/Lbm752/2014__H5N6
A/Environment/Guangdong/40929/2015__H5N6
A/Syrrhaptes_Paradoxus/Guangdong/Zh283/2015__H5N6
A/Environment/Guangdong/33311/2015__H5N6
A/Duck/Guangdong/04.15_Szbaxq021/2015__H5N6
A/Environment/Shenzhen/3/2015__H5N6
A/Guangxi/55726/2016__H5N6
A/Chicken/Guangdong/Fg594/2015__H5N6
A/Environment/Guangxi/44389/2015__H5N6
A/Environment/Guangxi/42586/2015__H5N6
A/Environment/Chongqing/45349/2015__H5N6
A/Environment/Chongqing/45134/2015__H5N6
A/Environment/Chongqing/45185/2015__H5N6
A/Environment/Chongqing/99178/2014__H5N6
A/Duck/Eastern_China/S0908/2014__H5N6
A/Duck/Jiangxi/95/2014__H5N6
A/Environment/Hunan/98885/2014__H5N6
A/Environment/Guangdong/79478/2014__H5N6
A/Guangdong/99710/2014__H5N6
A/Duck/Laos/Xby004/2014__H5N6
A/Chicken/Shenzhen/2396/2013__H5N6
A/Duck/Guangzhou/018/2014__H5N6
A/Chicken/Shenzhen/1061/2013__H5N6
A/Environment/Hunan/07767/2015__H5N6
A/Duck/Wuhan/Jxyfb22/2015__H5N6
A/Duck/Sichuan/Ncxj15/2014__H5N6
A/Sichuan/26221/2014__H5N6
A/Duck/Jiangxi/13469/2014__H5N6
A/Environment/Guangxi/03218/2012__H5N1
A/Duck/Vietnam/Ncvd-1940/2012__H5N1
A/Duck/Hunan/S4220/2011__H5N1
A/Guangxi/1/2009__H5N1
A/Hubei/1/2010__H5N1
A/Duck/Jiangsu/K1203/2010__H5N8
A/Anhui/1/2005__H5N1
A/Goose/Shantou/753/2002__H5N1
A/Chicken/Hong_Kong/Fy64w/2010__H9N2
A/Chicken/Guangdong/Zcy/2011__H9N2
A/Chicken/Jiangsu/Q3/2010__H9N2
A/Chicken/Jiangxi/18513/2014__H7N9
A/Yunnan/0127/2015__H5N6
A/Environment/Guangdong/40113/2015__H5N6
A/Shenzhen/1/2015__H5N6
A/Chicken/Zhejiang/C1/2013__H9N2
A/Chicken/Qingdao/009/2014__H9N2
A/Chicken/Zhejiang/Hj/2007__H9N2
H5N1
H9N2
0.02
